# Developing a consensus patient safety curriculum for junior doctors in Taiwan: a modified Delphi protocol

**DOI:** 10.1136/bmjopen-2026-123020

**Published:** 2026-10-22

**Authors:** Kun-Jen Yang, Selma Omer, Ben Jackson, P Vivekananda-Schmidt

**Affiliations:** 1 School of Medicine and Population Health, The University of Sheffield, Sheffield, UK; 2 Department of Emergency Medicine, Chang Gung Memorial Hospital in Chiayi, Chiayi, Taiwan

**Keywords:** MEDICAL EDUCATION & TRAINING, Quality in health care, Patient Care Management, Delphi Technique, Research Design

## Abstract

**Abstract:**

**Introduction:**

While international frameworks guide patient safety education, their impact depends on local training structures and sociocultural context. In Taiwan, patient safety training has not yet been consolidated into a coherent curriculum, and hierarchical, blame-oriented norms may discourage incident reporting and learning. The aim of this research programme includes establishing expert consensus on the core content for a Taiwan-contextualised patient safety curriculum for junior doctors through an adapted Delphi study.

**Methods and analysis:**

This protocol outlines an online modified Delphi study with three phases: (1) document analysis of international frameworks to draft curriculum statements; (2) patient and public involvement and engagement to refine statement relevance and clarity; and (3) a multiround Delphi survey. Phases I and II were completed as preparatory phases to develop the Delphi questionnaire for use in Phase III. Phase III is underway, and we are inviting 80–100 multidisciplinary experts in Taiwan, anticipating 11–30 respondents in the final round. Expert panellists are rating statements with free-text comments. Between rounds, controlled feedback will provide summary statistics and analysis of free-text comments to inform subsequent ratings. Consensus was prespecified as median ≥4 and IQR ≤1, supported by ≥70% of respondents rating 4–5. If a consensus is not reached, a further round will be conducted. Free-text data will be analysed qualitatively to support interpretation and implementation considerations.

**Ethics and dissemination:**

Ethics approvals for the consensus survey were obtained in the UK and Taiwan. Outputs will include endorsed curriculum content and implementation insights to guide future curriculum development. Findings will be disseminated through peer-reviewed publication and conferences.

**STRENGTHS AND LIMITATIONS OF THIS STUDY**

- To our knowledge, this is the first study to establish expert consensus on the core content of a contextually adapted patient safety curriculum for junior doctors in Taiwan, and it may offer a useful model in settings facing similar educational and sociocultural challenges.
- The study triangulates international patient safety frameworks, patient and public involvement and engagement (PPIE) and local clinical expertise to support a curriculum that is both globally informed and locally relevant.
- The online modified Delphi process enables anonymous participation across different geographical regions and institutions, helping to reduce the influence of professional hierarchy in the Taiwanese medical context.
- Although the study aims to recruit a multidisciplinary panel, participants may not fully represent all relevant perspectives across Taiwan’s healthcare and medical education systems.

## Introduction

 Patient safety, defined as the avoidance of unintended or unexpected harm during the provision of healthcare,^1^ is a core priority for healthcare systems. According to the WHO, patient harm is the 14th leading cause of the global burden of disease.^2^ Globally, around 1 in 10 hospitalised patients experience harm related to healthcare, and approximately half of these events are considered preventable.^3^ Importantly, unsafe care is not simply the result of individual mistakes, but is shaped by broader system factors and the underlying safety culture.^4 5^ Improving patient safety therefore requires structured education that builds healthcare professionals’ knowledge, shared language and practical skills in communication, teamwork and human factors, while fostering systems thinking and a strong safety culture.^3–5^

Over the past decades, several international organisations have proposed structured frameworks for patient safety education, including those developed by the WHO, the National Health Service (NHS) and the Accreditation Council for Graduate Medical Education and ACGME International (ACGME(-I)).^5–7^ These frameworks have helped standardise core topics and competencies. However, their impact depends on how well they align with local training structures and sociocultural contexts. Patient safety education should also connect with standards that shape how these competencies are applied and sustained in clinical practice. Internationally, accreditation systems such as Joint Commission International incorporate patient safety requirements into organisational standards,^8^ while in Taiwan, national patient safety goals set by the Ministry of Health and Welfare include priorities such as teamwork and effective communication, safety culture and incident management.^9^ Aligning educational content with such practice-oriented standards may help bridge formal training and the application of patient safety principles in clinical practice after graduation.

Junior doctors are a key target group because they work on the clinical frontline during a transition towards independent practice and often undertake high-volume, high-risk tasks.^10–12^ They tend to report fewer incidents,^13^ and may be particularly vulnerable to medication-related errors^14^ and handover-related communication failures.^15^

In Taiwan, hierarchical norms and a blame-oriented climate may also discourage speaking up, incident reporting and learning from error, thereby limiting opportunities for system improvement.^16–18^ Doctors accounted for only 1.2% of submitted incident reports, compared with 68.9% submitted by nurses.^19^ Against this background, patient safety training has not yet been consolidated into a coherent curriculum, and delivery may vary across institutions. Structured patient safety education can improve learners’ knowledge, attitudes and safety-related behaviours.^20^ Strengthening patient safety competencies, including speaking up and incident reporting, may therefore provide a practical entry point for organisational learning and cultural change.

Before developing a patient safety curriculum for junior doctors, consensus is needed on its essential content. This should be informed by international frameworks and evidence-based best practices, while also reflecting Taiwan’s contextual barriers to ensure credibility and feasibility. This protocol describes an online modified Delphi study to establish expert consensus on the core content of a Taiwan-contextualised patient safety curriculum for junior doctors. Its anonymity and iterative feedback can reduce hierarchy effects and support participation from geographically dispersed experts.^21 22^ Although the curriculum will be specific to Taiwan, the study may also illustrate how international patient safety frameworks can be adapted to local sociocultural and organisational contexts.

### Aim

To establish expert consensus on the core content of a Taiwan-contextualised patient safety curriculum for junior doctors.

## Objectives

1. To generate Taiwan-contextualised patient safety curriculum statements for junior doctors.
2. To establish expert consensus on these statements through Delphi rounds and thereby define the core curriculum content.
3. To identify key implementation barriers and enablers for this consensus-based curriculum in Taiwan, and to explore expert views on feasible technology-enhanced learning approaches to support delivery and engagement of the curriculum.

## Methods and analysis

### Study overview

Our approach is informed by Cumyn and Harris’ process for content validation of curriculum consensus guidelines.^23^ We adapted its key principles: content delineation, stakeholder-informed review through patient and public involvement and engagement (PPIE), and iterative consensus-building into an online modified Delphi design. The protocol is reported in accordance with the ACcurate COnsensus Reporting Document (ACCORD) guideline for consensus methods.^24^ This study comprises three phases: Phase I, content delineation; Phase II, PPIE; and Phase III, an online modified Delphi process. Phases I and II commenced in September 2025 and have been completed as preparatory phases to develop and refine the Delphi questionnaire for Phase III. Phase III is now using this questionnaire to establish expert consensus. The study was not prospectively registered. This protocol was submitted for peer-reviewed publication before completion of the Delphi consensus process.

### Phase I: content delineation (document analysis for development of Delphi statements)

Phase I delineated the likely core content of a Taiwan-contextualised patient safety curriculum for junior doctors. Draft Delphi statements were created through a structured document analysis of international frameworks to identify key competencies, values and learning priorities relevant to junior doctors.

We selected three widely used frameworks with explicit curricular domains and educational guidance: the WHO Patient Safety Curriculum Guide: Multi-professional Edition,^5^ the NHS Patient Safety Syllabus^6^ and the ACGME(-I) CLE Pathways to Excellence: Expectations to Achieve Safe and High-Quality Patient Care.^7^ Other frameworks were reviewed initially but not retained for the final comparison where their core concepts were already represented within the selected frameworks.^25 26^

We used structured document analysis to extract and synthesise curricular elements across the three frameworks. The WHO topics provided the initial organising structure, and content from the NHS and ACGME(-I) frameworks was mapped to the closest WHO topic, with additional categories created where needed. Extracted items were refined through de-duplication, consolidation and clarification to support Delphi rating. Initial extraction and synthesis were led by KJY, with iterative review by PVS, SO and BJ to enhance conceptual alignment, completeness and face validity. The synthesis matrix is provided in online supplemental file 1.

This synthesis informed the initial draft Delphi statements (V.1). In addition to framework synthesis, TEL statements were informed by an ongoing scoping review of technology-enhanced patient safety education for junior doctors, initiated during study preparation. Statements on barriers and enablers were informed by literature relevant to the Taiwanese context.^16 27^ Revisions across iterations were documented internally through statement versioning.

### Phase II: PPIE consultation and statement refinement

Phase II aimed to refine the draft Delphi statements through patient and public perspectives before expert consensus rating. Patient and public involvement can strengthen the relevance of patient safety policy and educational frameworks by incorporating perspectives grounded in lived experience.^5 28^ The aim of the PPIE consultation was to improve the statements using patient and carer perspectives.

Convenience sampling identified five Taiwanese public contributors with selection guided by recent or ongoing experience of receiving healthcare or supporting a family member receiving care within the Taiwanese healthcare system. The group included people with recurrent healthcare needs related to disability and carers with experience of chronic illness, critical care, end-of-life care and long-term disability-related care. Selection focused on obtaining varied lived experiences of healthcare rather than patient safety expertise. Contributors received the draft Delphi statements (V.1) in Mandarin in advance and were invited to provide any comments from a patient or carer perspective on aspects such as questionnaire length, unclear wording and any important issues missed. KJY then facilitated a brief online group consultation using the same broad prompts to encourage open discussion. KJY took notes during the discussion and used the main points to refine the Delphi statements.

The consultation raised several issues about questionnaire clarity and consistency. Some contributors were unclear about the scope of patient safety; they also pointed out differences in how statements were phrased. Two broader issues were also raised: communication with patients and families and the specific safety needs of vulnerable or high-risk patient groups. The resulting refinements and additions are summarised in table 1.

**Table 1:** Key PPIE feedback and resulting changes to the Delphi questionnaire

Key PPIE feedback	Resulting change
Unclear scope of patient safety and intended questionnaire respondents	Introductory instructions were clarified to better explain the purpose and target respondents of the Delphi questionnaire.
Inconsistent phrasing across statements	Statement openings were standardised from forms such as ‘Learners should…’ to ‘Patient safety education should…’ to improve consistency and clarity.
Communication with patients and families	New statement added: ‘Patient safety education should train learners in communication skills for engaging with patients and families, especially in sensitive or high-risk situations’.
Safety needs of vulnerable/high-risk patient groups	New statement added: ‘Patient safety education should prepare learners to identify and respond appropriately to the needs of high-risk patient populations, such as the elderly, children or patients with disabilities’.

PPIE, patient and public involvement and engagement.

The revised statement set (V.2) was then pilot-tested with six senior medical educators to check comprehensibility, completion time and usability of the online questionnaire. They were selected because of their experience in postgraduate medical education. Pilot responses were not included in the Delphi consensus analysis. Their feedback informed minor wording and formatting refinements, resulting in the final statement set (V.3), which is now being used in Phase III. The questionnaire is provided in online supplemental file 2.

### Phase III: modified Delphi (consensus process)

Phase III is the main consensus phase of the study and commenced in August 2026. An online modified Delphi process, using a predefined set of statements developed through framework synthesis, PPIE and pilot testing, is being used to establish expert consensus on the draft patient safety curriculum statements. The panel comprises multidisciplinary experts in Taiwan, including medical educators, patient safety and quality leaders, healthcare administrators and senior clinicians (eg, attending physicians and senior nurses). All have relevant experience in patient safety, medical education or training governance.

Panel eligibility requires either (1) at least 5 years of clinical practice including experience in patient safety and/or medical education; or (2) at least 3 years of substantive involvement in patient safety, healthcare quality, medical education or training governance. These criteria are intended to ensure sufficient professional experience to provide informed judgements on patient safety curriculum content. They also allow the panel to include a range of perspectives from clinical practice, education and governance. 5 years of clinical practice was considered likely to reflect sustained exposure to healthcare systems and patient safety issues in practice, whereas the alternative criterion of 3 years of substantive involvement allows inclusion of individuals with focused expertise in relevant leadership, educational or quality-improvement roles.

Delphi studies generally do not have a fixed panel size, and panel size is commonly determined by study purpose and the practical feasibility of iterative participation.^29 30^ However, panels of 11–30 participants have been described as a typical and workable range for expert consensus studies.^31^ Approximately 80–100 experts are being invited, anticipating attrition across rounds and aiming to retain 11–30 respondents in the final round.

KJY is responsible for identifying eligible participants and coordinating recruitment. Potential participants are identified through purposive sampling and snowball referrals via professional networks and relevant organisations, such as hospital patient safety units and postgraduate training leads. Invitations are sent by email with a link to the online survey. The supporting framework synthesis is not provided separately to panellists to reduce the potential influence of established international frameworks on their ratings.

#### Round 1 (initial rating)

In round 1, participants rate each statement on a 5-point Likert scale (1=strongly disagree to 5=strongly agree) and are invited to provide free-text comments for each section. Each statement is presented in both Mandarin and English.

After Round 1, the study team will compile structured controlled feedback for each statement, including the group median, IQR and the proportion of respondents selecting the top two categories (4–5), alongside an anonymised summary of key qualitative comments. Controlled feedback will be provided between rounds, following published Delphi guidance^29^ and Delphi reporting recommendations (CREDES).^21^ In subsequent rounds, participants will be shown this group feedback and will have access to their previous ratings to support re-rating. Minor wording refinements may be made between rounds to improve clarity and reduce ambiguity while preserving the underlying construct. All revisions will be documented in an audit trail.

#### Round 2 (re-rating and consensus refinement)

In Round 2, participants will review the collated feedback and re-rate all statements. The purpose of Round 2 is to support convergence of views across the full statement set. Free-text responses will remain available to allow participants to explain their ratings, suggest wording improvements and identify any issues related to clarity, feasibility or contextual relevance.

#### Round 3 (optional final confirmation)

A third Delphi round will be conducted only if, after Round 2, a small number of statements do not meet the prespecified consensus criteria and ratings remain dispersed. If required, Round 3 will focus solely on re-rating these specific statements with the same controlled feedback to seek final confirmation. This optional round is intended to confirm the panel’s position on borderline statements rather than to revisit the full statement set.

### Consensus definition

Consensus criteria were defined in advance. A statement will be considered endorsed for inclusion if it achieves a median ≥4 and IQR ≤1, supported by ≥70% of respondents rating the statement in the top two categories (4–5). These criteria were selected to reflect both the level and consistency of agreement and were informed by published Delphi guidance.^21 22 29^ Statements meeting these criteria after Round 2 (or Round 3, if conducted) will be endorsed for inclusion in the proposed consensus curriculum. Statements that do not meet the consensus criteria will be reported transparently. Where a statement does not reach consensus, rating patterns will be used to describe the disagreement. Qualitative comments, where available, will help interpret the findings and inform future refinement.

### Data collection and analysis

All Delphi rounds are administered online using QuestionPro. Only submitted responses will be included in the analyses. Participants who complete a round will be invited to subsequent rounds. Response rates and attrition will be monitored across rounds.

Free-text comments will be analysed qualitatively to support statement refinement and to synthesise implementation considerations. Comments will be collated by statement and section and coded into concise categories, including suggestions on clarity and wording, reasons for rating and practical feasibility considerations. KJY will draft the summaries and proposed wording changes, which will be reviewed by the research team. Key points will be summarised anonymously within controlled feedback in subsequent rounds.

Each Delphi round will remain open for approximately 1 month. A reminder email will be sent 1 week after the initial invitation to participants who have not yet responded, with a final reminder sent shortly before the round closes.

Within the Delphi consensus process, the research team does not participate in ratings or override consensus decisions.

### Patient and public involvement

Patient and public contributors were involved during Phase II to help refine the draft Delphi statements before expert rating. Further details are provided in the Phase II section. PPIE contributors will not participate in the Delphi expert rating rounds, and a summary of the study findings will be shared with them following study completion.

## Ethics and dissemination

Ethical approval was obtained from the University of Sheffield Research Ethics Committee (reference: 240179874) and the Institutional Review Board of Chang Gung Memorial Hospital, Chiayi, Taiwan (IRB No.: 202401395B0).

Recruitment is restricted to closed professional channels, including individual professional emails, official institutional channels and private professional groups. The survey is not posted on publicly accessible social media to reduce the risk of participation by ineligible individuals. Potential participants receive a participant information sheet and may contact the researcher with any questions before deciding whether to participate.

At the start of each round, participants will complete a mandatory online consent page before accessing the survey. Participation is voluntary, and participants may withdraw at any time before submitting their responses for the round.

Survey responses will be anonymised to other panel members, and only aggregated results and anonymised summaries of comments will be shared as controlled feedback. Contact details will be used solely for survey distribution and follow-up across rounds and will be stored separately from response data. Any information collected to administer the honorarium will also be stored separately from survey responses. Participants will receive an honorarium of New Taiwan dollar (NTD) 400 (approximately £10) per completed round as a token of appreciation for their time and contribution.

Findings from this study will be disseminated through peer-reviewed publication and conference presentations. The results will be used to inform the development of a Taiwan-contextualised patient safety curriculum for junior doctors and may subsequently support future continuing professional development. The findings will also be shared with relevant national patient safety and healthcare quality organisations, including the Joint Commission of Taiwan, to support dialogue and future alignment between patient safety education, evolving national patient safety priorities and clinical practice. An accessible summary of the findings will also be shared with the patient and public contributors involved in Phase II. Where appropriate, their reflections on the findings may be sought to support interpretation.

## Discussion

This protocol describes a staged approach to establishing expert consensus on the core content of a Taiwan-contextualised patient safety curriculum for junior doctors. A key strength is the integration of international guidance with local patient, public and professional perspectives rather than the direct adoption of an existing framework. This approach is relevant to others seeking to contextualise international patient safety guidance.

The preparatory phases serve complementary purposes. Phase I provides conceptual breadth and traceability through the synthesis of international frameworks, while Phase II incorporates PPIE before expert rating. PPIE can help identify issues that may be overlooked from professional perspectives.^28^ In this study, it informed improvements in questionnaire clarity and consistency and identified two relevant areas insufficiently represented in the initial statements: communication with patients and families and the safety needs of vulnerable or high-risk patient groups. Phase III then uses iterative anonymous rating and controlled feedback to establish multidisciplinary expert consensus. Prespecifying the consensus criteria and providing structured feedback between rounds also help make the Delphi process clearer and more consistent.^21 22^

The consensus findings will subsequently inform curriculum development and evaluation with junior doctors. Education alone cannot create a strong patient safety culture, as organisational and system factors also influence safe practice.^4 5^ Nevertheless, structured education can strengthen knowledge, attitudes and safety-related behaviours and may provide a foundation for wider organisational change.^20^

The PPIE offers both strengths and limitations. While incorporating PPIE into modified Delphi studies can represent a strength in health research,^32^ we acknowledge that in this instance, the PPIE consultation was relatively small and achieved through convenience sampling. Although the PPIE contributors brought varied healthcare experiences, their perspectives cannot represent the full diversity of patients and the public in Taiwan.

However, a smaller number of PPIE participants facilitated meaningful discussions and depth that is more challenging with a larger group.^33 34^ The research team agreed five participants was proportionate to the purpose of the exercise as the purpose of PPIE was not consensus but consultation to refine wording, identify missed aspects on the statements and increase relevance for all stakeholders. Additionally, the consensus statements did not entirely rely on the PPIE exercise but were derived from the literature evidence.

Reporting direct quotes from the PPIE discussion would also have strengthened the reporting, but the consultation was not recorded and only meeting notes were available. Researcher positionality may have influenced the initial framing of statements, although framework synthesis and PPIE were used to mitigate this. In addition, expert recruitment through professional networks and snowball sampling may not capture the full range of professional and institutional perspectives across Taiwan.

Finally, Delphi consensus represents expert agreement on curriculum priorities rather than evidence of educational or clinical effectiveness. The resulting consensus may not fully align with international patient safety frameworks or national priorities in Taiwan.^9^ These need to be the focus of next steps once a consensus statement is derived. Future piloting and evaluation will be required to assess the feasibility and educational value of the curriculum.

## Supplementary material

10.1136/bmjopen-2026-123020 online supplemental file 1

10.1136/bmjopen-2026-123020 online supplemental file 2
