# Supplementary material for: Developing a consensus patient safety curriculum for junior doctors in Taiwan: a modified Delphi protocol
Source: BMJ Open. 2026 Oct 22;16(10):e123020. doi: 10.1136/bmjopen-2026-123020 (PMC13691782; doi:10.1136/bmjopen-2026-123020)
Supplement: Supplementary file 1 [file bmjopen-2026-123020.reviewer_comments.pdf]

PEER REVIEW HISTORY

BMJ Open publishes all reviews undertaken for accepted manuscripts. Reviewers are asked to complete a checklist review form and are provided with free text boxes to elaborate on their assessment. These free text comments are reproduced below.

ARTICLE DETAILS

Title (Provisional)

Developing a consensus patient safety curriculum for junior doctors in Taiwan: a modified Delphi protocol

Authors

Yang, Kun-Jen; Omer, Selma; Jackson, Ben; Vivekananda-Schmidt, P

VERSION 1 - REVIEW

**Reviewer**	**1**
**Name**	**Kovacs Burns, Katharina**
**Affiliation**	**University of Alberta, Health Sciences**
**Date**	**31-Jul-2026**
**COI**	**None**

Thank you for your paper outlining the protocol to establish expert consensus on core patient safety statements which will define a patient safety curriculum – the study when completed will be a great asset to the curriculum for junior doctors in Taiwan but will also be of interest to many other medical programs in other countries. Patient safety seems to have dropped off the radar or been significantly under emphasized in many of our education and training programs over the years, as evidenced by the escalation in patient safety incidents worldwide.

Regarding the paper – there are a few areas of the paper which need some additional details. The following comments or suggestions are provided to strengthen the paper:

• Your literature search is appropriate for focusing on the patient safety curriculum, but there should also be a link to patient safety training and practices following graduation of doctors and other professionals, many of which will be aligned with standards or accreditation in health care systems and specifically hospitals or other care settings. The paper would be strengthened to include the link with your local as well as global or other patient safety standards/accreditation groups that support the practices needed to ensure patient safety education and training continue with the hope of improving patient safety outcomes. The evidence based standards these groups develop help guide practices and these should align with education curricula for doctors and others. Hence the need to

provide that connection in this paper either in the introduction and/or in the dissemination or conclusion section – at least indicate sharing or aligning patient safety competencies with the standards and sharing your findings with those who develop and review standards on a regular basis to ensure updates and current practices. Some of these key global groups include the Joint Commission International (JCI), DNV International Accreditation Standards, ISQua External Evaluation Association. Some counties also are worth looking at – e.g. Accreditation Canada Health Standards Organization and their Patient Safety guidelines called Required Safety Practices. In Taiwan, you have the Joint Commission of Taiwan, National Patient Safety Goals supported through the Ministry of Health and Welfare and other systems. It's worth bridging in some of this to support the curriculum and skills/practices you are proposing which will lead to better informed and skilled doctors who hopefully will transfer their education about patient safety and sustain their knowledge and skills in their practices.

• Some sections in the paper can be reordered for a smoother flow – i.e. the Patient and pubic involvement paragraph on page 10 of 23 would be better embedded in the Phase II section where you first mention the PPIE group. And the section on the expert panel eligibility and recruitment would be better inserted within the first paragraph under Phase III section where you first mention this panel group. This realignment would improve the continuity of the information.

• Regarding patient and public involvement – it seems you brought in this group to do separate pre-testing of the survey to inform or refine statements. And although the rationale was good, the description of the methodology and the results needs more details to show the value of having this group involved in the survey refinement. The issue or question for me starts with the selection of the patients/public and why only five. This seems very inadequate as was the criteria used to select them, which was that they had experience being cared for in the health system. Were there other criteria for their selection? This is missing and or lacks rigor – e.g. what was their reading/literacy level to ensure they could read and understand the statements they were asked to review? What experience did the patients have with patient safety or patient safety incidents which is the real focus of the statements and not just patient care experience? How were the questions worded for them to be able to discuss statement clarity, potential bias, blind spots and any patient priorities they felt needed to be addressed? If none of the patients had any patient safety incident experiences, how would they know what else was missing? All of these criteria impact the type of patient you then have and the results of the discussion. This seems like a limitation of the study and should be noted as such.

• As there is no real results section in this paper, the results given for the patient and public involvement in phase II need to stand out more. Right now the results are high level and general – more details about the actual discussion results based on the questions asked are needed (hence the previous suggestion to provide more details about the discussion questions etc. related to Phase II). We should know what the five patients/public individuals

said relative to the questions asked of them. These details would provide the evidence to support changes in the statements or develop the new ones – e.g. Were there any quotes that stood out from the discussion to support the changes in statements or adding new ones? More details also support the importance or value of having this group involved in the survey development.

• There is no discussion section – would be good to pull pieces together, supported by the literature where possible, but then leading to the next steps for implementing Phase III and what follows from there. The limitations need to be described in this section as well.

• The Ethics and dissemination section should be divided. Ethics is usually a stand alone section, and the dissemination piece should really be part of your discussion or conclusion where you indicate the steps in completing the Delphi process and follow up with your proposed plan for the dissemination of findings, especially with the medical programs. What other follow up, if any, would you recommend or are planning after this study?

I hope these comments are helpful and can strengthen your paper. Even noting any limitations will not take away from your study, but it's important to include.

Thank you again!

VERSION 1 - AUTHOR RESPONSE

Response to Reviewer

We would like to thank the reviewer for the careful and constructive comments on our manuscript. We have revised the manuscript accordingly and provide our point-by-point responses below. For clarity, several longer reviewer comments have been divided into their main points. The wording has been condensed without changing the substance of the comments.

Comment 1: Link with patient safety standards and accreditation

The reviewer suggested strengthening the link between patient safety education and the standards or accreditation systems that shape clinical practice after graduation. In particular, the reviewer recommended:

(a) linking the proposed curriculum with relevant international patient safety/accreditation standards;

(b) linking it with Taiwanese patient safety standards and national priorities; and

(c) indicating how the findings may be shared with relevant organisations to support future alignment between education and practice.

Response:

Thank you for this helpful suggestion. We have added a clearer link between patient safety education and standards used in clinical practice. In the Introduction, we now refer to Joint Commission International (JCI) as an international example and to Taiwan's national patient safety goals set by the Ministry of Health and Welfare. We have also added examples of relevant national priorities, including teamwork and communication, safety culture and incident management.

We have also expanded the dissemination section to state that the findings will be shared with relevant national organisations, including the Joint Commission of Taiwan.

Comment 2: Reorder sections

The reviewer suggested reordering two sections to improve continuity:

(a) integrating the detailed PPIE description into Phase II, where PPIE is first introduced; and

(b) moving the expert panel eligibility and recruitment information to the beginning of Phase III, where the panel is first described.

Response:

Thank you for this suggestion. We have moved the detailed PPIE information into Phase II and shortened the separate Patient and Public Involvement section to avoid repetition. We have also moved the expert panel eligibility and recruitment information to the beginning of Phase III, as suggested.

Comment 3: More detail on PPIE methods and participant selection

The reviewer requested further methodological detail about the PPIE consultation, particularly:

(a) the rationale for involving five contributors and how they were selected;

(b) their relevant healthcare or patient safety experience;

(c) whether literacy or ability to understand the statements was considered;

(d) how the consultation questions/prompts were framed; and

(e) acknowledgement of relevant limitations of the PPIE approach.

Response:

Thank you for this suggestion. We agree that the manuscript will benefit from the additional details as suggested. We have added these details to Phase II and included

the main limitations in the Discussion.

The PPIE was a focused consultation to help refine statements that had already been developed from patient safety frameworks and the literature. It was not intended as a representative survey or consensus exercise. The five contributors were recruited using convenience sampling through the researcher's network with selection guided by recent or ongoing experience of healthcare in Taiwan, either as patients or carers. We have added more information about the range of healthcare experiences represented in the group.

We did not formally assess health literacy or require previous experience of a recognised patient safety incident. Contributors received the statements in Mandarin in advance and were able to review them. We were interested in broader patient and carer experience, not only experiences involving a recognised safety incident.

We have also described the broad prompts used during the consultation. In the Discussion, we now consider both the strengths and limitations of having a small PPIE group. We also acknowledge recruitment through the research's network and the use of meeting notes without audio-recording as limitations.

Comment 4: Make the PPIE findings more visible

The reviewer asked for the outcomes of the PPIE consultation to be reported more clearly, including:

(a) more detail on the main feedback raised by contributors;

(b) clearer evidence showing how this feedback led to statement refinement or the addition of new statements; and

(c) where appropriate, illustrative examples of contributor feedback to demonstrate the value of PPIE in questionnaire development.

Response:

Thank you for this suggestion. We have expanded the Phase II section to give more detail about what was raised during the PPIE consultation and how this affected the questionnaire.

Contributors raised questions about the scope of patient safety and commented on the way some statements were phrased. They also raised two areas that were not sufficiently covered in the original draft: communication with patients and families, and the safety needs of vulnerable or high-risk patient groups.

We have added a table showing the main feedback and the changes made in response. This includes clarification of the introductory instructions, more consistent wording across statements and the addition of two new statements on communication and

high-risk patient groups.

We did not include direct quotes because the consultation was not recorded. The reported feedback is therefore based on notes taken during the discussion, and this is also acknowledged as a limitation.

The two new statements added following the consultation were:

“Patient safety education should train learners in communication skills for engaging with patients and families, especially in sensitive or high-risk situations.”

“Patient safety education should prepare learners to identify and respond appropriately to the needs of high-risk patient populations, such as the elderly, children, or patients with disabilities.”

Comment 5: Add a Discussion

The reviewer recommended adding a Discussion section to:

(a) bring together the rationale and contribution of the different study phases;

(b) support the discussion with relevant literature where possible;

(c) outline the next steps following Phase III; and

(d) describe the study limitations.

Response:

Thank you for this suggestion. We have added a Discussion section that brings together the three phases of the study and places the approach within the patient safety and Delphi literature.

The Discussion also describes what will follow the Delphi study. The consensus findings will be used to inform curriculum development and piloting for junior doctors, while recognising that education is only one part of improving patient safety.

We have also added the main study limitations. These include the small PPIE group, recruitment through researcher’s networks, and the use of meeting notes without audio-recording. We also acknowledge that the initial statements were partly shaped by researcher judgement. Finally, Delphi consensus will show agreement on curriculum priorities but will not show whether the curriculum will be educationally or clinically effective.

Comment 6: Ethics and dissemination

The reviewer suggested separating the Ethics and Dissemination sections and

expanding the description of dissemination and planned follow-up after completion of the Delphi study.

Response:

Thank you for this suggestion. Following the editor's guidance, we have kept Ethics and dissemination as a combined section in line with the journal format. However, we have added more detail about dissemination and follow-up after the Delphi study.

The findings will be used to inform the development of a Taiwan-contextualised patient safety curriculum for junior doctors and may also support future continuing professional development. We have added that the findings will be shared with relevant national organisations, including the Joint Commission of Taiwan. An accessible summary will also be shared with the patient and public contributors involved in Phase II.

The new Discussion also outlines the planned next steps, including curriculum development, piloting and future evaluation.

Additional changes

We also made several minor revisions throughout the manuscript for clarity and consistency, including updating the tense to reflect the current status of Phase III and correcting minor wording.

We have also replaced Supplementary File 2 with the ethics-approved version of the Delphi questionnaire that is being used in Phase III. The previously submitted file contained some later wording refinements, but these did not change the meaning of the statements or the additions made following the PPIE consultation.

VERSION 2 - REVIEW

**Reviewer**	**1**
**Name**	**Kovacs Burns, Katharina**
**Affiliation**	**University of Alberta, Health Sciences**
**Date**	**24-Sep-2026**
**COI**	

Thank you for the revisions to the protocol paper. The revisions including additional descriptions improve the overall paper. There are just a couple of suggestions I would make that would improve the paper further:

One suggestion is to move the section on Patient and Public Involvement under Phase II so it is aligned more appropriately for the reader. As it is, this section seems to be inappropriately placed.

The second suggestion is for the authors to have a thorough edit of the paper for spelling typos and grammar checks.

Thank you again for pulling this protocol together. The results from the Phase III Delphi rounds will be very interesting. Best of luck!
