# Supplementary material for: Developing a consensus patient safety curriculum for junior doctors in Taiwan: a modified Delphi protocol
Source: BMJ Open. 2026 Oct 22;16(10):e123020. doi: 10.1136/bmjopen-2026-123020 (PMC13691782; doi:10.1136/bmjopen-2026-123020)
Supplement: Supplementary file 2 [file bmjopen-16-10-s001.docx]

**Supplementary File 1: Phase I framework mapping matrix**

This supplementary file summarises the framework mapping and synthesis undertaken in Phase I to support development of the initial Delphi statements, including:

(A) a brief overview of the three source frameworks, and

(B) the matrix used to compare the WHO, NHS and ACGME(-I) frameworks and identify common and distinct areas relevant to statement development.

| Part A: Overview of source frameworks | | | |
| --- | --- | --- | --- |
|  | WHO | NHS | ACGME(-I) |
| Framework title | Patient Safety Curriculum Guide: Multi-professional Edition | National Patient Safety Syllabus | International Clinical Learning Environment Pathways to Excellence: Expectations to Achieve Safe and High-Quality Patient Care |
| Target audience | Global healthcare educators and students across professions | All NHS staff | Trainees and educators in graduate medical education (GME) |
| Purpose | To support effective capacity building in patient safety education and prepare learners for safe practice | To strengthen patient safety education and training across the NHS | To support discussion and improvement in clinical learning environments for safe, high-quality patient care |
| Distinctive contribution | Emphasises capacity building and the hidden curriculum | Emphasises national standardisation and staged capability development | Provides a vision framework for safe, high-quality clinical learning environments |
| Major topics or domains | 1. Topic 1: What is patient safety 2. Topic 2: Why applying human factors is important for patient safety 3. Topic 3: Understanding systems and the effect of complexity on patient care 4. Topic 4: Being an effective team player 5. Topic 5: Learning from errors to prevent harm 6. Topic 6: Understanding and managing clinical risk 7. Topic 7: Using quality improvement methods to improve care 8. Topic 8: Engaging with patients and carers. 9. Topic 9: Infection prevention and control 10. Topic 10: Patient safety and invasive procedures   Topic 11: Improving medication safety | 1. Domain 1: Systems approach to patient safety 2. Domain 2: Learning from incidents 3. Domain 3: Human factors, human performance, and safety management 4. Domain 4: Creating safe systems 5. Domain 5: Being sure about safety | 1. Patient safety (PS) 2. Health Care Quality (HCQ) 3. Teaming (TM) 4. Supervision (SV) 5. Well-Being (WB) 6. Professionalism (PROF) |
|  |  |  |  |
| Part B: Cross-framework synthesis matrix | | | |
|  | WHO mapping | NHS mapping | ACGME(-I) mapping |
| Introduction | T1 | D1 | PS1  PS2 |
| Human factors | T2 | D3  D5 | WB4 |
| Systems thinking | T3 | D1  D4 | PS1c |
| Teamwork and Teaming | T4 | D3 | TM |
| Incident learning and error management | T5 | D2 | PS3  PS4 |
| Clinical risk management | T6 | D2  D4  D5 | PS2b |
| Quality improvement | T7 | D3  D4 | HCQ |
| Patient and Carer Engagement | T8 | D1.S5 | PS6  TM3  PROF4 |
| Infection | T9 | Not explicitly categorised | Not explicitly categorised |
| Invasive procedures | T10 | Not explicitly categorised | SV5a |
| Medication safety | T11 | D1.S5.3 | Not explicitly categorised |
| Well-being | Not explicitly categorised | D3 | WB |
| Proactive safety and resilience | Not explicitly categorised | D1.S3 | PS1 |
| Professionalism and medicolegal aspects | Not explicitly categorised | D1.S6 | PROF |
| Clinical supervision | Not explicitly categorised | Not explicitly categorised | SV |
|  |  |  |  |

**Abbreviations**

WHO = World Health Organization; NHS = National Health Service; ACGME(-I) = Accreditation Council for Graduate Medical Education (International); T = WHO topic; D = NHS domain; S = NHS sub-domain; PS = Patient Safety; HCQ = Health Care Quality; TM = Teaming; SV = Supervision; WB = Well-Being; PROF = Professionalism.

**Coding note**

WHO topics are shown as T1–T11. NHS domains and sub-domains are shown as D and S codes where applicable. For ACGME(-I), abbreviations such as PS, HCQ, TM, SV, WB, and PROF refer to the six focus areas within the framework, while accompanying numbers or letters (e.g., PS1, WB4, SV5a) indicate the closest mapped pathway within that focus area.
