# Supplementary material for: Developing a consensus patient safety curriculum for junior doctors in Taiwan: a modified Delphi protocol
Source: BMJ Open. 2026 Oct 22;16(10):e123020. doi: 10.1136/bmjopen-2026-123020 (PMC13691782; doi:10.1136/bmjopen-2026-123020)
Supplement: Supplementary file 3 [file bmjopen-16-10-s002.docx]

**Supplementary File 2: Phase III Delphi questionnaire**

Part A: Background of participants

| Email  For sending follow-up survey rounds and reminders only.  電子郵件  僅用於寄送後續問卷與提醒 |  |
| --- | --- |
| Please confirm that you meet the eligibility criteria for participation in this study:   1. At least 5 years of clinical experience; or 2. At least 3 years of experience in patient safety or medical education.   請確認您符合本研究之參與條件:  1. 至少有5年臨床工作經驗, 或  2. 至少有3年病人安全或醫學教育經驗 | |
| Total years of professional experience (healthcare, patient safety, and/or medical education)  專業工作(醫療專業/病人安全/醫學教育)總計年資 | □ 5-9年 (5-9 years)  □ 10-14年 (10-14 years)  □ 15-19年 (15-19 years)  □ 20-24年 (20-24 years)  □ 25年以上 (25 years or more) |
| Role  角色 | ☐ Postgraduate clinical teacher (畢業後臨床教師) ☐ University faculty (大學教師) ☐ Patient safety officer (病人安全官) ☐ Hospital administrator/manager (醫院主管) ☐ Other, please specify: _________ (其他，請說明) |
| Institution  服務機構 | ☐ Educational institution /University (學校教育機構) ☐ Patient safety organisation /association (病人安全相關協會) ☐ Medical centre (醫學中心) ☐ Regional hospital (區域醫院) ☐ District hospital (地區醫院)  ☐ Local clinic (診所)  ☐ Government health authority(政府衛生主管機關) ☐ Other, please specify: _________ (其他，請說明) |
| Primary professional background  主要專業背景 | ☐ Medicine (醫學)  ☐ Nursing (護理)  ☐ Pharmacy (藥學)  ☐ Allied health profession(醫事專業人員)  ☐ Healthcare administration or management(醫療相關行政或管理)  ☐ Education(教育)  ☐ Other, please specify: _________ (其他，請說明) |

Part B: Delphi statements

| **Section 1: Core Patient Safety Topics (第一部分：病人安全核心主題)** | |
| --- | --- |
| This section focuses on the basic topics of patient safety. These topics were selected based on key domains identified in major international patient safety education frameworks. In Taiwan, these topics may or may not be clearly taught. Please rate the importance of the following topics based on your experience.  Scale: 1~5  1 = Not important at all / Strongly disagree  5 = Very important / Strongly agree  本部分聚焦於病人安全教育的基本主題。這些主題是根據多份國際病人安全教育框架所歸納出的核心領域所制定。在台灣，這些主題可能尚未被明確教導。請根據您的經驗評估下列主題的重要性。  量表：1～5  1 = 完全不重要 / 非常不同意  5 = 非常重要 / 非常同意 | |
| T01 | Patient safety education should emphasize the value of psychological safety and open disclosure in clinical environments.  病人安全教育應強調臨床環境中心理安全與開放揭露的重要性。 |
| T02 | Patient safety education should provide scenarios for identifying latent risks related to fatigue, distractions, and process failures.  病人安全教育應提供情境案例以辨識疲勞、分心及流程失誤等潛在風險。 |
| T03 | Patient safety education should highlight interprofessional teamwork in preventing errors and ensuring continuity of care.  病人安全教育應強調跨專業團隊合作在預防錯誤與確保照護連續性的重要性。 |
| T04 | Patient safety education should train learners to use structured communication tools (e.g., SBAR, I-PASS) during clinical handoffs and emergencies.  病人安全教育應訓練學員在臨床交班與緊急情境中使用結構化溝通工具（如 SBAR、I-PASS）。 |
| T05 | Patient safety education should train learners in communication skills for engaging with patients and families, especially in sensitive or high-risk situations.  病人安全教育應訓練學員與病人及家屬的溝通技巧，特別是在敏感或高風險的情境中。 |
| T06 | Patient safety education should help learners recognize common clinical errors (e.g., prescribing, communication) and suggest appropriate, context-specific prevention strategies.  病人安全教育應協助學員辨識常見臨床錯誤（如開立處方、溝通錯誤），並提出適切、具情境性的預防策略。 |
| T07 | Patient safety education should include proactive risk identification strategies and tools (e.g., safety walkrounds, checklists, huddles).  病人安全教育應包含積極性風險辨識策略與工具（如安全巡視、檢查清單、確認會議）。 |
| T08 | Patient safety education should include the purpose and process of reporting systems.  病人安全教育應涵蓋通報系統的目的與運作流程。 |
| T09 | Patient safety education should introduce learners to basic quality improvement (QI) cycles (e.g., PDSA) and tools (e.g., run charts, flow maps) to analyze and improve care processes.  病人安全教育應介紹學員基本的品質改善循環（如 PDSA）與工具（如趨勢圖、流程圖），以分析並改善照護流程。 |
| T10 | Patient safety education should prepare learners to involve patients and families in safety discussions and shared decision-making.  病人安全教育應訓練學員讓病人與家屬參與安全相關討論與共同決策。 |
| T11 | Patient safety education should prepare learners to identify and respond appropriately to the needs of high-risk patient populations, such as the elderly, children, or patients with disabilities.  病人安全教育應訓練學員識別並妥善回應高風險病人族群的需求，例如高齡者、兒童或有障礙的病人。 |
| T12 | (Open-ended prompt)  Are there any additional patient safety topics that you believe should be prioritized in Taiwan’s PGY curriculum?  開放式問題：您認為還有哪些病人安全主題應優先納入台灣 PGY 課程中？ |
| T13 | (Open-ended prompt)  In this section, are there any statements that you think should be combined or split, or any wording that is unclear or not appropriate to the Taiwanese context? Please indicate the relevant item number(s) and suggest alternative wording. Your feedback will be used anonymously to revise and refine these statements for inclusion in the next round of the survey.  開放式問題：在本部分中，是否有任何敘述應合併或拆分，或有任何用字不清楚、不適合臺灣情境之處？請指出相關題號，並請建議替代文字。您的意見將匿名協助修訂與精煉這些敘述，並納入下一輪問卷。 |
|  |  |
| **Section 2: Technology-Enhanced Learning (第二部分：科技增能學習)** | |
| Technology-enhanced learning (TEL) is an emerging delivery method in medical education. However, the potential of TEL in patient safety education remains underexplored. Please rate the importance of each of the following TEL approaches in effectively supporting patient safety education.  Scale: 1~5  1 = Not important at all / Strongly disagree  5 = Very important / Strongly agree  科技增能學習（TEL）是醫學教育中新興的教學方式，但其在病人安全教育中的應用潛力仍有待深入探討。請根據您的經驗，評估下列各種 TEL 方法在促進病人安全教育方面的重要性。  量表：1～5 1 = 完全不重要 / 非常不同意 5 = 非常重要 / 非常同意 | |
| L01 | Online modules can effectively improve foundational knowledge in patient safety.  線上模組可有效提升學員對病人安全的基礎知識。 |
| L02 | Simulation (including low-fidelity and ward-based drills) can effectively enhance non-technical skills in safety-critical scenarios, such as communication, teamwork, and managing distractions.  模擬訓練（包含低擬真度模擬與病房情境演練）可有效提升學員於高風險情境下的非技術性能力，如溝通、團隊合作與分心管理。 |
| L03 | Game-based approaches (e.g., escape rooms, serious board games) can foster active participation and improve error identification and team coordination in patient safety education.  遊戲化教學方式（如密室逃脫、病安桌遊）可提升學員參與度，並增進錯誤辨識與團隊協作能力。 |
| L04 | Embedding structured reflection and feedback into TEL activities can support attitude and behavior change.  在 TEL 活動中融入結構化反思與回饋，能促進態度與行為的改變。 |
| L05 | Low-cost tools (e.g., comics, case-based discussions) are practical for high-pressure healthcare environments.  低成本工具（如漫畫、案例討論）適合在高壓的醫療環境中應用。 |
| L06 | (Open-ended prompts)  Based on your experience, are there other digital or TEL strategies you would recommend for patient safety education?  開放式問題：根據您的經驗，您是否建議其他可用於病人安全教育的數位或科技增能學習方式？ |
| L07 | (Open-ended prompts) Following your response to the previous question, please explain your reasons. You may wish to consider factors such as cost, time, faculty capacity, and learner engagement. Please also suggest any other TEL strategies not listed above.  承上題，原因為何？可考量成本、時間、師資量能與學習者參與度等因素。也請提出上述未列出的其他 TEL 策略建議。 |
|  |  |
| **Section 3: Barriers and Enablers (第三部分：推動障礙與促進因素)** | |
| There are different barriers and enablers to implement patient safety education in Taiwan. Here are some statements about the barriers and enablers. Please rate the level of agreement for each of the following statements. 1 = Not important at all / Strongly disagree  5 = Very important / Strongly agree  在推動病人安全教育的過程中，台灣的臨床環境可能面臨不同的阻礙或促進因素。以下是一些相關陳述，請評估您對每一項陳述的同意程度。  1 = 非常不同意 / 完全不重要  5 = 非常同意 / 非常重要 | |
| B01 | A lack of trained faculty is a major barrier to implementing patient safety education.  缺乏受過訓練的教學人力是推動病人安全教育的重要障礙。 |
| B02 | Hierarchical culture and fear of blame are significant barriers to error reporting during training.  階層文化與對責備的恐懼是訓練期間阻礙錯誤通報的主要因素。 |
| B03 | The use of institutionally endorsed and standardized teaching materials and schedules would promote consistency in patient safety education delivery and support its long-term sustainability.  採用機構認可且標準化的教學教材與課程安排，有助於確保病人安全教育的一致性與長期永續推行。 |
| B04 | Visible leadership support and role modeling are key enablers of safety culture development.  領導階層的支持與身教示範是建立病人安全文化的重要促進因素。 |
| B05 | Linking patient safety education to formal assessments (e.g., PGY evaluation) may improve learner motivation and accountability.  將病人安全教育納入正式評量（如 PGY 評估）可能有助於提升學員的學習動機與責任感。 |
| B06 | (Open-ended prompt)  What institutional or cultural factors have most affected patient safety training in your clinical setting?  開放式問題：在您的臨床工作環境中，哪些制度或文化因素最影響病人安全訓練的推行？ |
| B07 | (Open-ended prompt)  Following your response to the previous question, what practical and feasible strategies could be used to address these barriers?  承上題，有哪些實際可行的策略可以因應這些障礙？ |
|  |  |
